# Leveraging PET to image folate receptor α therapy of an antibody-drug conjugate

**DOI:** 10.1186/s13550-018-0437-x

**Published:** 2018-08-28

**Authors:** Christian Brand, Ahmad Sadique, Jacob L. Houghton, Kishore Gangangari, Jose F. Ponte, Jason S. Lewis, Naga Vara Kishore Pillarsetty, Jason A. Konner, Thomas Reiner

**Affiliations:** 1 Department of Radiology, Memorial Sloan Kettering Cancer Center, 1275 York Avenue, New York, NY 10065 USA; 2 Department of Chemistry, Hunter College and PhD Program in Chemistry, The Graduate Center of the City University of New York, New York, NY USA; 3 ImmunoGen, Inc, Waltham, MA USA; 4 Molecular Pharmacology Program, Memorial Sloan Kettering Cancer Center, New York, NY USA; 5 Department of Radiology, Weill Cornell Medical College, New York, NY 620 USA; 6 Department of Medicine, Memorial Sloan Kettering Cancer Center, New York, NY USA

**Keywords:** ^89^Zr, Companion diagnostic, PET imaging, Antibody-drug-conjugate

## Abstract

**Background:**

The folate receptor α (FRα)-targeting antibody-drug conjugate (ADC), IMGN853, shows great antitumor activity against FRα-expressing tumors in vivo, but patient selection and consequently therapy outcome are based on immunohistochemistry. The aim of this study is to develop an antibody-derived immuno-PET imaging agent strategy for targeting FRα in ovarian cancer as a predictor of treatment success.

**Methods:**

We developed [^89^Zr]Zr-DFO-M9346A, a humanized antibody-based radiotracer targeting tumor-associated FRα in the preclinical setting. [^89^Zr]Zr-DFO-M9346A’s binding ability was tested in an in vitro uptake assay using cell lines with varying FRα expression levels. The diagnostic potential of [^89^Zr]Zr-M9346A was evaluated in KB and OV90 subcutaneous xenografts. Following intravenous injection of [^89^Zr]Zr-DFO-M9346A (~90 μCi, 50 μg), PET imaging and biodistribution studies were performed. We determined the blood half-life of [^89^Zr]Zr-DFO-M9346A and compared it to the therapeutic, radioiodinated ADC [^131^I]-IMGN853. Finally, in vivo studies using IMG853 as a therapeutic, paired with [^89^Zr]Zr-DFO-M9346A as a companion diagnostic were performed using OV90 xenografts.

**Results:**

DFO-M9346A was labeled with Zr-89 at 37 °C within 60 min and isolated in labeling yields of 85.7 ± 5.7%, radiochemical purities of 98.0 ± 0.7%, and specific activities of 3.08 ± 0.43 mCi/mg. We observed high specificity for binding FRα positive cells in vitro. For PET and biodistribution studies, [^89^Zr]Zr-M9346A displayed remarkable in vivo performance in terms of excellent tumor uptake for KB and OV xenografts (45.8 ± 29.0 %IA/g and 26.1 ± 7.2 %IA/g), with low non-target tissue uptake in other organs such as kidneys (4.5 ± 1.2 %IA/g and 4.3 ± 0.7 %IA/g). A direct comparison of the blood half life of [^89^Zr]Zr-M9346A and [^131^I]-IMGN853 corroborated the equivalency of the radiopharmaceutical and the ADC, paving the way for a companion PET imaging study.

**Conclusions:**

We developed a new folate receptor-targeted ^89^Zr-labeled PET imaging agent with excellent pharmacokinetics in vivo. Good tumor uptake in subcutaneous KB and OV90 xenografts were obtained, and ADC therapy studies were performed with the precision predictor.

**Electronic supplementary material:**

The online version of this article (10.1186/s13550-018-0437-x) contains supplementary material, which is available to authorized users.

## Background

Over the last years, the development of novel antibody-drug conjugates (ADCs) has been a trending topic in cancer therapy [1]. This technology uses an antibody to deliver a cytotoxic drug selectively to a tumor cell population by targeting tumor-associated receptors. There are several reasons driving the development of antibody-drug conjugates for cancer treatment. One feature is that a humanized or human antibody bears very high specificity and affinity towards its cancer specific antigen [2]. Another feature is that some of the most potent non-targeted chemotherapeutics such as maytansine have extensive side effects in patients [3], which intuitively could be reduced by targeting the drugs to their intended site of action. However, the transition of antibody-drug conjugates into the clinic would benefit greatly from non-invasive precision predictors that could potentially allow for patient stratification and early evaluation of effectiveness. Patient stratification according to the expression of membrane-bound antigens typically requires invasive biopsy via needle aspiration of multiple tissue regions to overcome tumor heterogeneity. In addition, decisions regarding patient management are often made by using archived biopsy results that do not necessarily reflect antigen expression at the time of treatment.

**Fig. 1:**
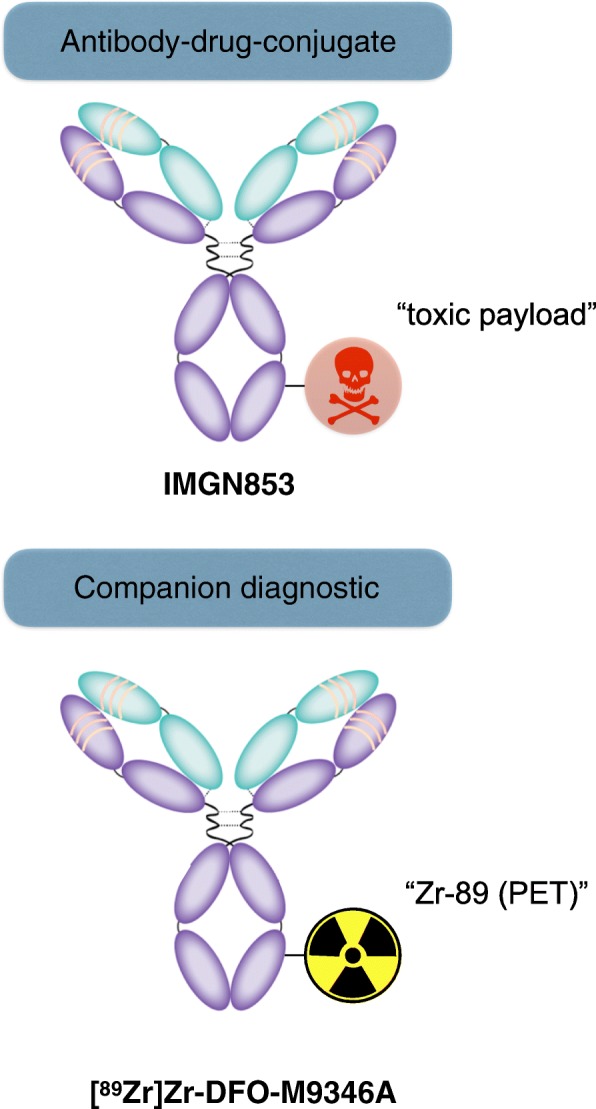
Concept of an antibody-based companion diagnostic for cancer therapy using positron emission tomography (PET) imaging. The humanized antibody, M9346A, functions as targeting vector for antibody-drug-conjugate as well as companion diagnostic

Mirvetuximab soravtansine (IMGN853) is an antibody-drug immunoconjugate that consists of a humanized monoclonal antibody (M9346A) targeting folate receptor alpha (FRα)-positive cancer cells [4] attached to a highly potent cytotoxic maytansinoid, DM4 (Fig. 1) [5]. Although IMGN853 has been evaluated in a phase 1 expansion clinical study [6, 7] and is currently being evaluated in the Forward1 (NCT02631876) phase 3 clinical study, it is noteworthy that IMGN853 is not the only folate receptor targeting therapeutic approach currently evaluated in clinical trials [8, 9]. Nonetheless, the antibody, with its high specificity and affinity towards FRα, has limited distribution in normal human tissue [10, 11] and mainly targets the overexpression in epithelial ovarian cancer and non-small cell lung cancer [12, 13]. After endocytosis, the ADC releases its toxic payload inside the cell via lysosomal degradation of the antibody and disulfide reduction of the sulfo-SPDB linker. Following binding of DM4 to the microtubules, the targeted cancer cell undergoes mitotic arrest and cell death.

In order to improve the drug development process of novel therapeutic agents, it is becoming more and more important to concurrently develop companion diagnostics [14–16]. With this in mind, the aim of this study was to develop and evaluate an ^89^Zr-labeled antibody derived from the FRα-targeting antibody, M9346A, as an immuno-positron emission tomography (immuno-PET) companion diagnostic agent for the antibody-drug conjugate IMGN853 that is based on the same humanized antibody (Fig. 1). In order to develop a M9346A-based PET imaging agent, we asked the following questions: (1) Is it possible to achieve the immunoreactivity after modification with desferrioxamine (DFO) as a chelator and radiolabeling with Zr-89? (2) Is this antibody-based PET imaging agent able to delineate ovarian cancer xenografts? (3) Does the pharmacokinetics of the radiopharmaceutical match with the pharmacokinetic profile of the antibody-drug-conjugate? (4) Can we predict antibody-drug conjugate success using the novel companion diagnostic?

## Methods

### Materials

Unless otherwise stated, all chemicals and solvents were used without further purification. Water used for this study was ultrapure (> 18.2 MΩcm^−1^ at 25 °C). Phosphate-buffered saline (PBS) as well as cell growth medium was purchased from the Media Preparation Facility at Memorial Sloan Kettering Cancer Center (MSK) (New York, NY). Humanized monoclonal antibody recognizing FRα and M9346A, as well as antibody-drug-conjugate IMGN853 were provided by ImmunoGen, Inc. (Waltham, MA) and further purified via a PD10 desalting column (GE Healthcare). Concentrations of solutions containing antibody were determined by using a NanoDrop™ 2000 spectrophotometer from Thermo Fisher Scientific (Waltham, MA). The bifunctional chelator *p*-isothiocyanatobenzyl-desferrioxamine (DFO-Bz-NCS) was purchased from Macrocycles (Plano, TX). ^89^Zr-oxalate was acquired from 3D Imaging, LLC (Maumelle, AR). HPLC reactions (1.0 mL/min, phosphate-buffered saline) were performed on a Shimadzu UFLC HPLC system equipped with a DGU-20A degasser, a SPD-M20A UV detector, a LC-20AB pump system, and a CBM-20A communication BUS module using a size exclusion column (GE Superdex™ 200, 10/300 GL).

### Functionalization and radiolabeling of antibody M9346A

The humanized antibody, M9346A, was functionalized with the bifunctional chelator *p*-isothiocyanatobenzyl-desferrioxamine (DFO-Bz-NCS) with a 1:3.5 antibody:DFO ratio, as previously described [17, 18]. Specifically, a solution of previously purified antibody M9346A (500 μL, 2.27 mg) in PBS was adjusted to pH = 8.5 with 1 M Na_2_CO_3_ (1.0 μL). Then, a solution of DFO-Bz-NCS (4.2 mM, 4.0 μL) in dimethylsulfoxide was slowly added and the reaction mixture was incubated at 37 °C for 90 min. Afterwards, the DFO-modified antibody was purified using a PD-10 desalting column and isolated in PBS solution for in vitro and in vivo studies.

A solution of ^89^Zr-oxalate in 1 M oxalic acid (5.0 μL, 85.1 MBq, 2.3 mCi) was added to phosphate-buffered saline (100 μL), and a 1 M sodium carbonate solution (4.8 μL) was used to adjust the pH to 7.1–7.4. Then, a solution of DFO-M9346A (500 μL, 500 μg) was added to the neutralized Zr-89 solution and the reaction mixture was incubated at 37 °C for 1 h. After purification using PD-10 desalting columns (GE Healthcare), [^89^Zr]Zr-DFO-M9346A (85.7 ± 5.7% isolated radiochemical yield and 3.08 ± 0.43 mCi/mg specific activity) was isolated in a PBS solution (2.0 mL) and radiochemical purity (98.0 ± 0.7%) was determined through radio-instant thin/layer chromatography. Then, the resulting ^89^Zr-radiolabeled antibody solution was diluted with PBS to the desired volume for in vitro and in vivo studies.

### Cancer cell lines

We chose four cancer cell lines with variable FRα expression levels to correlate imaging findings. The HeLa-derived cancer cell line KB (high expression), the ovarian cancer cell line OV90 (low/medium expression), the human lung cancer cell line H2110 (low/medium), and the human lung cancer cell line A549 (low/no expression) were purchased from ATCC (Manassas, VA). No further cell line authentication was conducted, and the cell lines were expended by passaging 2–3 times, aliquoted, and frozen in liquid nitrogen. For use in in vitro as well as in vivo experiments, the cell lines were grown in medium recommended by American Type Culture Collection (ATCC) and passaged regularly at 70–80% confluence every 3–4 days. All cell lines were cultured at 37 °C and 5% carbon dioxide.

### Characterization and in vitro experiments

[^89^Zr]Zr-DFO-M9346A was investigated for stability by incubating the radioligand in 1% bovine serum albumin at 37 °C for 3 days. Therefore, radiochemical purity of [^89^Zr]Zr-DFO-M9346A was tested at various time points via radio-instant thin layer chromatography (Eckert & Ziegler, Germany) with ethylendiaminetetraacetic acid (50 mM) as mobile phase.

A Lindmo assay [19] was performed to validate the integrity of the ^89^Zr-radiolabeled antibody M9346A. In detail, serial dilutions of KB cells were incubated with one concentration of [^89^Zr]Zr-DFO-M9346A in Eppendorf vials (1.5 mL) on a shaking rack. After incubation at room temperature for 1 h, cells were washed with ice-cold PBS and the retained radioactivity on cells was measured using a WIZARD^2^ automatic γ-counter from PerkinElmer.

Furthermore, an in vitro binding assay was performed with cell lines expressing different levels of FRα, as previously described [20]. One day prior to the in vitro experiment, the cells lines KB, OV90, and H2110, as well as A549 (each 1.0 × 10^6^ cells per well, > 90% viability for each cell line) were seeded into 6-well plates containing growth medium (2.0 mL) and incubated at 37 °C to form subconfluent cell monolayers. Then, growth medium was removed from the 6-well plates, cells were washed with PBS, and 900 μL of growth medium was added. After 1 h of incubation at 37 °C, a solution of [^89^Zr]Zr-DFO-M9346A (0.5 μCi, 100 ng) in PBS (100 μL) was added to the wells. For blocking studies, cells were pre-incubated with humanized antibody M9346A (10 μg) 5 min prior to the addition of ^89^Zr-radiolabeled antibody (0.5 μCi, 100 ng). To determine the number of cells at the time of the experiment, a separate set (*n* = 3) of wells for each cell line and experiment was analyzed by detaching the cells with trypsin and counted immediately using a Vi-cell XR cell viability analyzer (Beckman Coulter). After 1 h post-incubation at 37 °C, the supernatant of each well was collected together with washing solution of ice-cold PBS. Then, cells were lysed with sodium hydroxide solution (1 M, 1.0 mL) for 5 min at room temperature. Finally, each cell suspension was collected and all radioactive samples were measured using a WIZARD^2^ automatic γ-counter from PerkinElmer (Waltham, MA). Receptor-specific uptake was determined by correlating cell-bound activity relative to non-bound activity in the supernatant and displayed as a percentage of applied activity per 5.0 × 10^5^ cells.

### Animals

All in vivo studies and procedures were performed in accordance with an approved protocol from the Institutional Animal Care and Use Committee at MSK. All in vivo experiments were carried out in female, athymic nude mice (Envigo; outbread; 6–8 weeks, 20–25 g). Subcutaneous KB and OV90 xenografts (150 ± 20 mm^3^) were established as previously described [5, 20]. In detail, suspended KB cells (1.5 × 10^6^, viability > 93%) or suspended OV90 cells (1.0 × 10^7^, viability: > 95%) in a solution containing a 1:1 mixture of Matrigel (Becton Dickinson, Bedford, MA) and cell culture media (no FBS for OV90 xenografts) were subcutaneously inoculated on the right shoulder of anesthetized mice (1.5–2.0% isoflurane (Baxter Healthcare) in medical air (2 L/min)). Prior in vivo studies, KB tumors were grown for 9–10 days post-implantation and OV90 tumors were grown for 21 days.

### In vivo PET/CT imaging and biodistribution

For in vivo studies, PET images were recorded using a small-animal Inveon® PET/CT system from Siemens (Knoxville, TN) and mice were anesthetized with 1.5–2.0% isoflurane at 2.0 L/min flow of medical air. PET images were analyzed using AsiPro VM™ software (Concorde Microsystems) and Inveon research workplace 4.1 software (Siemens Healthcare). For biodistribution studies, mice were euthanized at pre-determined time points through asphyxiation with carbon dioxide and organs of interest were collected, weighed, and counted using a WIZARD^2^ automatic γ-counter from PerkinElmer. The percentage of tracer uptake stated as percentage injected activity per gram of tissue (%IA/g) was calculated as the activity associated with tissue per organ weight per actual injected dose, decay corrected to the start time of counting. KB tumor-bearing mice (*n* = 10) were injected with [^89^Zr]Zr-DFO-M9346A (7.75 ± 0.25 MBq, 209.0 ± 6.7 μCi, 50 μg) in PBS (200 μL). PET images were acquired at 4 h, 24 h, 48 h, and 72 h post-injection, and biodistribution studies were performed at 24 h and 72 h (each cohort, *n* = 5). OV90 tumor-bearing mice (*n* = 6) were injected with [^89^Zr]Zr-DFO-M9346A (2.02 ± 0.08 MBq, 48.4 ± 2.0 μCi, 50 μg) in PBS (200 μL). PET images were acquired at 24 h and 48 h post-injection, and biodistribution studies were performed at 24 h and 48 h post-injection of radioligand. Each tumor-bearing cohort exhibited expected bone uptake upon release of Zr-89 from the chelator desferrioxamine (DFO) of about 5–10 %IA/g.

### Radiolabeling of ADC

All iodine-131 radiolabeling reactions were performed in pre-coated Iodogen (1,3,4,6-tetrachloro-3α,6α-diphenyl glycoluril) tubes (100 μg per tube). For instance, an aqueous solution of Na^131^I (37.0 MBq, 1.0 mCi) was added to a phosphate buffered saline solution of IMGN853 (250 μg, 45 μL) and the tube containing the reaction mixture was gently agitated at 1-min intervals at room temperature over a period of 10 min. After purification using PD-10 desalting columns, [^131^I]-IMGN853 (32.1 MBq, 0.87 mCi) was isolated in 87% radiochemical yield and high radiochemical purity (99.9%).

### Blood half-life studies

Female athymic nude mice (6–8 weeks, *n* = 5) were injected intravenously with [^89^Zr]Zr-DFO-M9346A (2.3 ± 0.2 MBq, 62.6 ± 4.6 μCi, 25 μg) or [^131^I]-IMGN853 (2.7 ± 0.1 MBq, 73.4 ± 2.6 μCi, 25 μg). At predetermined time points (0.5 h, 1.0 h, 3.0 h, 6.0 h, 24 h, 48 h, and 72 h), a sample of blood was obtained from the great saphenous vein of each animal in a heparin-coated capillary glass tube and the weights of the collected blood samples were obtained by using an analytical balance Toledo XS105 from Mettler. The radioactivity of the blood samples was recorded with a WIZARD^2^ automatic γ-counter from PerkinElmer. The residual radiotracer, expressed as a percentage injected dose per gram (%IA/g), was calculated as the activity present in the blood weighed per actual injected dose, decay corrected to the time of counting.

### Biodistribution studies

For biodistribution studies, OV90 tumor-bearing nude mice (6–8 weeks, *n* = 5) were injected intravenously with [^131^I]-IMGN853 (1.42 ± 0.02 MBq, 38.3 ± 0.4 μCi) in PBS (200 μL). At 48 h post-injection, mice were euthanized and organs of interest were collected, weighed, and counted with a WIZARD^2^ automatic γ-counter from PerkinElmer.

### In vivo therapy studies

Tumor volumes for all mice were measured via manual caliper measurements of the longest dimension (*x*), shortest dimension (*y*), and height (z), assuming an ellipsoid shape (*V* = (3.1415/6) × (x × y × z) [mm^3^]). During tumor measurements, weight for all mice was recorded, and after initial tumor measurements, mice were randomized into three cohorts (*n* = 3–5) per cohort ensuring all cohorts had tumor volumes of 150–200 mm^3^. One day after initial tumor volume measurements, mice in the therapy cohort A were co-administered with a solution of ADC (1.25 mg/kg, 31.2 μg) and companion imaging agent [^89^Zr]Zr-DFO-M9346A (3.34 ± 0.04 MBq, 90.3 ± 1.1 μCi, 25 μg) in PBS (200 μL). Cohort B was injected with the companion imaging agent [^89^Zr]Zr-DFO-M9346A (3.68 ± 0.07 MBq, 99.5 ± 1.8 μCi, 25 μg). Cohort C was injected with PBS (200 μL). Two days after administration, mice of cohort A and cohort B were anesthetized with 1.5–2.0% isoflurane at 2.0 L/min flow of medical air and PET/CT imaging was accomplished over 10 min using small-animal Inveon® PET/CT system from Siemens (Knoxville, TN). Then, tumor volumes were determined via caliper measurement every 3 to 4 days up to an endpoint volume of > 1000 mm^3^. Additionally, all mice were assessed twice per week throughout the study for outward signs of toxicity and decreasing body weight.

## Results

### Development of [^89^Zr]Zr-DFO-M9346A

**Fig. 2:**
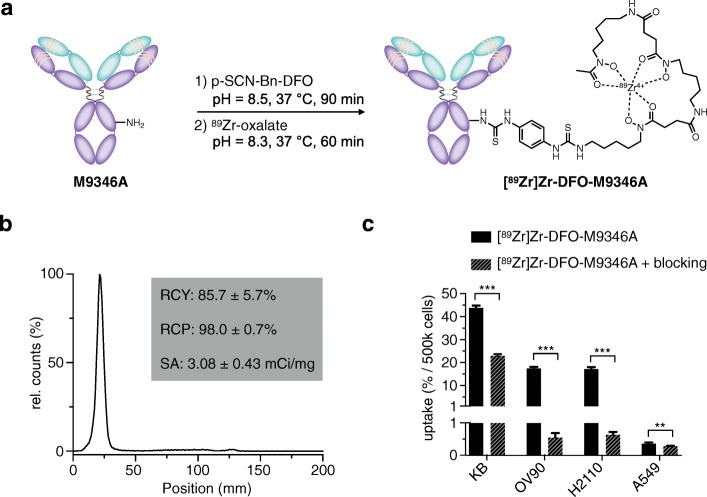
Synthesis and characterization of [^89^Zr]Zr-DFO-M9346A. **a** Conjugation of *p*-isothiocyanatobenzyl-desferrioxamine (DFO-Bz-NCS) to humanized antibody M9346A in basic aqueous conditions at 37 °C for 90 min and ^89^Zr-radiolabeling of DFO-M9346A at 37 °C for 60 min. **b** After purification of reaction mixture using a PD-10 desalting column, quality control of [^89^Zr]Zr-DFO-M9346A was performed using radio-instant thin layer chromatography in a solution of EDTA (50 mM). **c** In vitro uptake studies of either [^89^Zr]Zr-DFO-M9346A (100 ng) or a mixture of [^89^Zr]Zr-DFO-M9346A (100 ng) and M9346A (10 μg) using cancer cell lines KB, OV90, H2110, and A549 with various expression levels of FRα. **P < 0.01; ***P < 0.001. Error bars represent the SD

Modification of the humanized monoclonal antibody (mAb), M9346A, with the bifunctional chelator DFO-Bn-NCS was accomplished using a previously reported method (Fig. 2a, Additional file 1: Figure S1) [17, 18, 21]. First, desferrioxamine was coupled to lysine-NH_2_ groups of the parent antibody (1:3.5 antibody: DFO ratio) at pH = 8.5 over 90 min in phosphate buffered saline, followed by purification using PD-10 desalting columns. Subsequently, radiolabeling of the modified mAb with neutralized [^89^Zr]Zr-oxalic acid solution proceeded in PBS at 37 °C over 60 min, followed by purification using PD-10 desalting columns. DFO-M9346A was radiolabeled reliably with good specific activity (3.08 ± 0.43 mCi/mg) and radiochemical purity (98.0 ± 0.7%). Lindmo assays of the resulting radiolabeled antibody construct confirmed that the immunoreactivity of the construct was established through binding of the antibody to FRα in vitro (83.4 ± 3.5%, Additional file 2: Figure S2). Stability of [^89^Zr]Zr-DFO-M9346A was examined in bovine serum albumin at 37 °C over a period of 72 h and showed that more than 95% of the radioligand remained intact (Additional file 3: Figure S3). Further characterization of [^89^Zr]Zr-DFO-M9346A included an in vitro cell uptake assay using cancer cell lines with various expression levels of FRα (Fig. 2c). We observed highly specific cell-associated uptake and retention in KB, OV90, and H2110 after 1 h of incubation at 37 °C (43.9 ± 0.9%, 17.6 ± 0.5%, and 17.3 ± 0.8%, per 500,000 cells respectively), corresponding to their folate receptor expression levels [5]. The cancer cell line A549 with very low expression levels of FRα displayed negligible uptake (0.4 ± 0.1%).

### In vivo and ex vivo experiments with [^89^Zr]Zr-DFO-M9346A in subcutaneous epithelial cancer xenografts

**Fig. 3:**
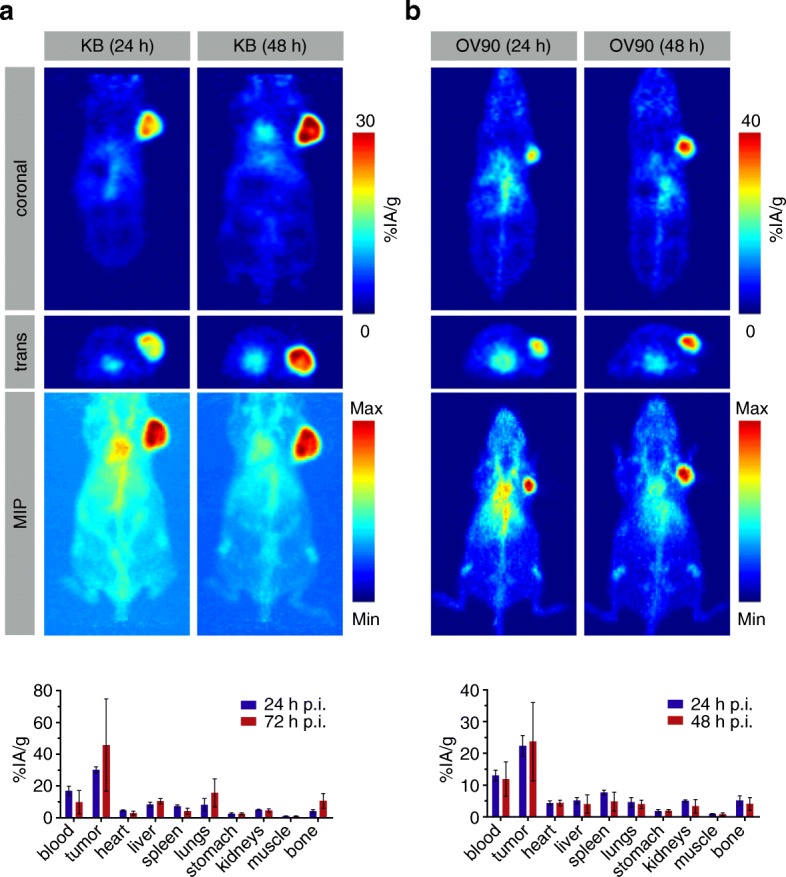
In vivo studies of [^89^Zr]Zr-DFO-M9346A: **a** PET images acquired at 24 h and 48 h post-injection and biodistribution studies performed at 24 h and 72 h with [^89^Zr]Zr-DFO-M9346A intravenously administered in KB tumor bearing mice. **b** PET images acquired at 24 h and 48 h post-injection and biodistribution studies performed at 24 h and 72 h with [^89^Zr]Zr-DFO-M9346A intravenously administered in tumor bearing mice

We first asked whether [^89^Zr]Zr-DFO-M9346A can target a tumor in vivo known to be high in expression of FRα (Fig. 3a and Additional file 4: Figure S4). After tail vein injection of the radioligand, small-animal PET imaging (4 h, 24 h, 48 h, and 72 h) and biodistribution studies (24 h and 72 h) were conducted using KB tumor-bearing mice (female, athymic, *n* = 10). Serial PET imaging showed clear delineation of the tumor already after 24 h with low uptake in normal tissue. The maximum intensity projections (MIPs) indicated that blood-pool and background activity cleared over time, leading to improved tumor-to-background ratios at 48 h post-injection. Ex vivo biodistribution data corroborated the PET data, as high tumor localization of [^89^Zr]Zr-DFO-M9346A at 24 h (30.1 ± 1.9 %IA/g, *n* = 5) was observed and tumor uptake increased over time out to 72 h (45.8 ± 29.0 %IA/g, *n* = 5) post-injection, suggesting an optimal time point for future PET imaging studies without increasing bone uptake over time (Additional file 5: Figure S5).

After the first proof-of-concept experiment using KB cells, we set out to validate [^89^Zr]Zr-DFO-M9346A in a more clinically relevant cancer model using the ovarian cancer cell line OV90. To this end, [^89^Zr]Zr-DFO-M9346A was intravenously injected into female athymic nude mice bearing a subcutaneous OV90 xenograft on the right shoulder. PET imaging and ex vivo biodistribution after 24 h and 48 h confirmed efficient retention of the radioligand in the tumor (24.2 ± 6.3 %IA/g, *n* = 6) with very limited background uptake (Fig. 3b and Additional file 6: Figure S6).

### Pharmacokinetic head-to-head comparison between IMGN853 and [^89^Zr]Zr-DFO-M9346A

**Fig. 4:**
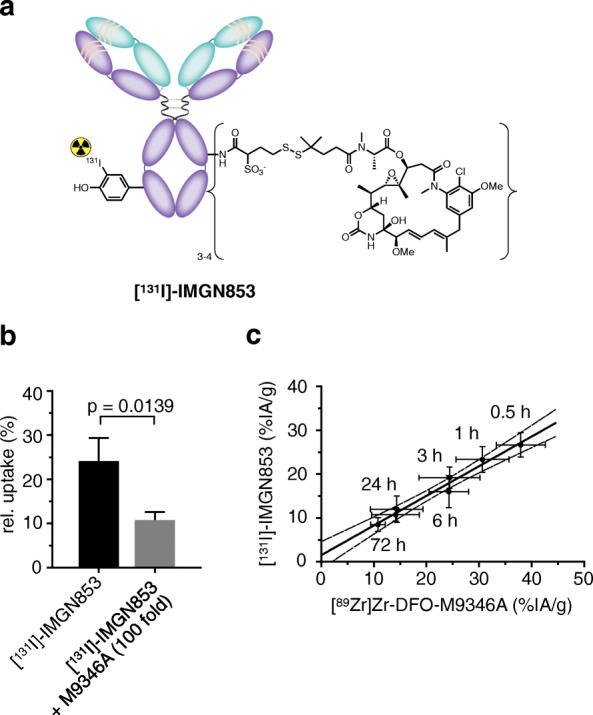
Pharmacokinetic head-to-head comparison between antibody-drug-conjugate, IMGN853 and companion diagnostic, [^89^Zr]Zr-DFO-M9346A. **a** Introduction of the radionuclide I-131 by direct halogenation of the antibody in the presence of Iodogen as chemical oxidant yielding radiolabeled antibody-drug-conjugate [^131^I]-IMGN853 (32.2 MBq, 0.87 mCi, 250 μg) in high radiochemical purity (> 98%). **b** In vitro characterization of [^131^I]-IMGN853. Incubation of KB cells with radiolabeled ADC or with a mixture of radiolabeled ADC (100 ng) and M9346A (10 μg). **c** Comparison of blood half-lives of [^131^I]-IMGN853 and [^89^Zr]Zr-DFO-M9346A (dashed line indicates the 95% confidence band)

After successful characterization of the antibody-based PET imaging agent showing very promising performance in vivo, we next set out to validate that the antibody-drug conjugate and companion diagnostic show nearly identical pharmacokinetic profiles. In order to achieve this goal, we performed a head-to-head comparison between both antibody constructs. In order to do that, we radiolabeled the antibody-drug conjugate, IMGN853, through direct halogenation with I-131 (Fig. 4a). [^131^I]-IMGN853 was isolated in good isolated radiochemical yields (86.7 ± 13.5%) and high radiochemical purity (99.3 ± 0.7%). Based on previous in vitro studies with [^89^Zr]Zr-DFO-M9346A, we tested whether the radiolabeled ADC retains its ability to bind to FRα and performed an in vitro uptake with using KB cells (Fig. 4b). Then, we went ahead and compared the drugs’ pharmacokinetic behavior by evaluating each tracer’s blood half-life in vivo. Figure 4c shows the correlation of tracer (%IA/g) in the blood at predetermined time points. The blood half-life for both radioligands was determined through serial bleeds in healthy mice, and the obtained data points were compared to each other. We found a good correlation between [^131^I]-IMGN853 and [^89^Zr]Zr-DFO-M9346A (*R*^2^ = 0.9736). Ex vivo biodistribution data revealed very similar biodistribution pattern in all organs compared to the companion diagnostic with slightly lower tumor uptake of [^131^I]-IMGN853 of 17.3 ± 5.2 %IA/g (Additional file 7: Figure S7).

### Leveraging PET imaging with [^89^Zr]Zr-DFO-M9346A during therapy with IMGN853

**Fig. 5:**
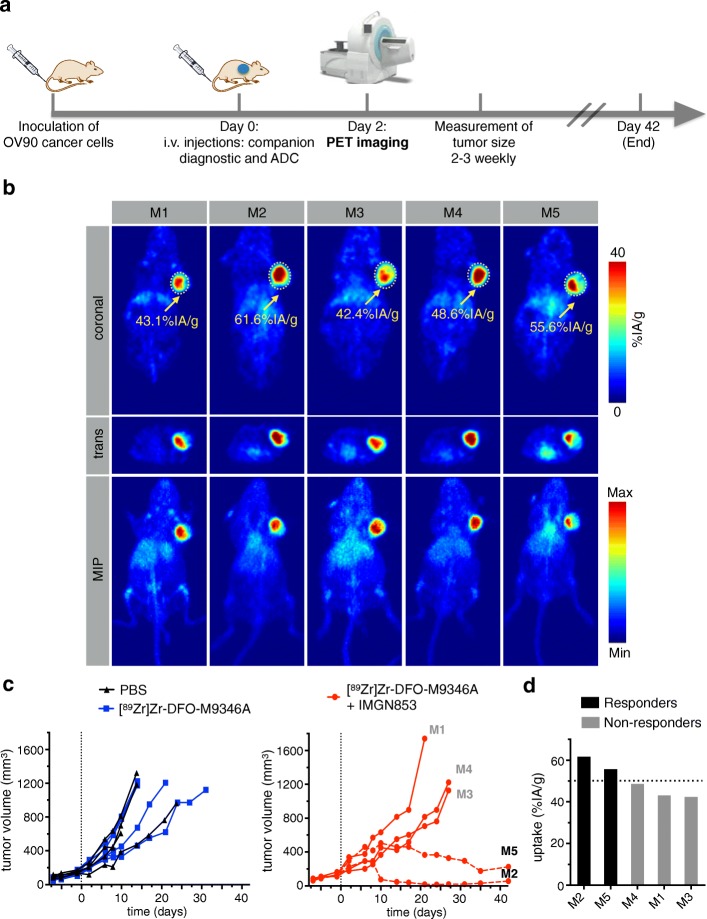
Therapy study with antibody-drug-conjugate, IMGN853, and companion diagnostic, [^89^Zr]Zr-DFO-M9346A, using OV90 xenografts. **a** Overall design of the therapy study including inoculation of cancer cells, injection of ADC and companion diagnostic, PET imaging after 2 days, and continuously measuring tumor growth. **b** PET images of mice injected with [^89^Zr]Zr-DFO-M9346A (90.3 ± 1.1 μCi, 25 μg) and IMGN853 (1.25 mg/kg, 31.2 μg) and quantification through ROI analysis of coronal slices. **c** Plot of the individual tumor volume post treatment (days). **d** Correlation of tracer uptake (%IA/g) and therapy outcome

Finally, we determined whether [^89^Zr]Zr-DFO-M9346A could be used as a companion diagnostic during ADC therapy to predict therapy outcome. For these experiments (Fig. 5a), mice bearing OV90 tumor xenografts were injected with an imaging dose of [^89^Zr]Zr-DFO-M9346A (90.3 ± 1.1 μCi, 25 μg) as well as with a therapy dose of IMGN853 (1.25 mg/kg, 31.2 μg). PET images were acquired 2 days post-administration, and tumor volume of each mouse was tracked over several days until the end of the study. In general, administration of [^89^Zr]Zr-DFO-M9346A during ADC treatment with IMGN853 confirmed good tumoral uptake with high tumor-to-tissue contrast (Fig. 5b). After quantification of the acquired PET images, we observed differences in tumoral uptake with the lowest uptake of 42.4 %IA/g and the highest of 61.6 %IA/g. Following the tumor volume of individual mice revealed a differential response in ADC treatment (Fig. 5c). At 6 weeks post-treatment, two mice of the therapy cohort receiving IMGN853 (1.25 mg/kg) responded by inhibited tumor growth, whereas three mice showed similar tumor growth curves as the cohorts injected with [^89^Zr]Zr-DFO-M9346A (positive control) and PBS (negative control). No weight losses were observed in any cohort of the therapy study (Additional file 8: Figure S8). Correlation between tumoral uptake (%IA/g) of [^89^Zr]Zr-DFO-M9346A and therapy outcome shows that in this cohort, mice with uptake greater than 50 %IA/g appeared to be responders and mice with lower than 50 %IA/g were non-responders (Fig. 5d). The cutoff level at 50 %IA/g was arbitrarily set and corresponds to the average tumor uptake of [^89^Zr]Zr-DFO-M9346A in this cohort.

## Discussion

Over the last decades, companion diagnostic agents have become more and more important in the development process of targeted drugs, particularly in oncology [14–16, 22]. Especially, nuclear medicine-based companion diagnostics targeting cancer-specific membrane bound receptors have received considerable attention [23–26]. In this study, we report a nuclear medicine-based companion diagnostic, [^89^Zr]Zr-DFO-M9346A, for the ADC mirvetuximab soravtansine (IMGN853). Currently, patient selections in clinical trials of IMGN853 are based on immunohistochemical (IHC) assessment of archival tumor tissue. Expression levels of FRα are not contemporaneous, and both intra- and inter-lesion tissue sampling is limited, making a non-invasive whole body imaging approach more favorable.

Following published protocols [17, 18], we were able to modify and radiolabel the FRα-targeting antibody M9346A in good radiochemical yields (88 ± 5%) and high-specific activities (3.08 ± 0.43 mCi/mg). [^89^Zr]Zr-DFO-M9346A was highly stable in human plasma (> 97% at 24 h). It also displayed relatively high immunoreactivity (> 80%) indicating conservation of the ability to bind to FRα in vitro. Furthermore, we observed high uptake in KB, OV90, and H2110 cells (43.9 ± 0.9%, 17.6 ± 0.5%, and 17.3 ± 0.8%, per 500,000 cells respectively). Co-incubation of OV90 and H2110 with a 100-fold excess of antibody M9346A further corroborates specific uptake. In case of the KB cancer cell line, incomplete blocking may be a result of the high abundance of FRα per cell in comparison to the other FRα-positive cancer cell lines [27]. Nevertheless, reduction in uptake was statistically significant (*p* < 0.001). Binding to A549 was negligible (< 1%), since FRα expression levels are reported to be low in comparison with other cancer lines.

Encouraged by the successful in vitro characterization of [^89^Zr]Zr-DFO-M9346A, we next sought to test the companion diagnostic in tumor-bearing mice. Initially, we injected the PET imaging agent in KB tumor bearing mice. Already 24 h after tracer administration, we observed good delineation of the tumor. According to the maximum-intensity-projection, slow blood clearance of the antibody construct caused accumulation in the heart region. However, qualitatively improved PET images were observed at 48 h and 72 h post-injection. Biodistribution data supported the results of the PET imaging studies, observing good anticipated tumor-to-blood ratios of 5.9 and tumor-to-bone ratios of 5.4 at 72 h post-administration of [^89^Zr]Zr-DFO-M9346A. In an OV90 tumor-bearing PET imaging study, we observed similar uptake patterns with good delineation of tumor and high imaging contrast. Based on the biodistribution studies, we reached slightly lower uptake of the companion diagnostic, which is likely due to the lower expression level of FRα in OV90 cells compared to KB cells.

We next wanted to set stage for being able to predict ADC therapy success using our new companion diagnostic. Both antibody-based constructs had similar pharmacokinetic profiles, similar to what we showed with a nanoparticle construct earlier [28]. Therefore, we were able to directly radiolabel the ADC with iodine-131. A standard in vitro uptake assay showed that binding as well as specificity of the antibody-based construct towards FRα was conserved, indicating minor modifications of the antibody. A direct comparison of the blood half-life of each radiolabeled antibody construct, [^131^I]-IMGN853 and [^89^Zr]Zr-DFO-M9346A, successfully indicated nearly identical pharmacokinetic behaviors in vivo. Radioiodination of antibodies with Iodogen is a standard method, suggesting that it is very unlikely that labeling conditions will lead to degradation of IMGN853. Since the antibody exhibits a more than 20-fold higher molecular weight in comparison with the added modifications (3–4 DM4 per antibody and 3–4 chelator per antibody), we expected minor alterations in the pharmacokinetics of each construct. However, other physiochemical properties such as polarity and charge of a molecule can influence drug pharmacokinetics as well.

We tested the ability of [^89^Zr]Zr-DFO-M9346A to predict FRα-targeted therapy with IMGN853 in vivo. The penultimate goal of this study was to distinguish between responders and non-responders at the beginning of a therapeutic intervention—which might be an additional first step to improve the interpretation of current and future clinical trials using IMGN853. Two days post-co-administration of [^89^Zr]Zr-DFO-M9346A and IMGN853 in OV90 tumor-bearing mice, we were able to delineate OV90 tumors, in which we observed heterogeneity of companion diagnostic, presumably dictated not only by target expression, but also tumor physiology [29]. By measuring the tumor volume of each animal over time, we were able to establish a threshold of tracer uptake at 50 %IA/g. A higher value indicated that the animal responded to the therapy, whereas a lower value indicated a non-responder.

## Conclusion

In summary, we have developed an antibody-based companion diagnostic measuring FRα expression in ovarian cancer during ADC therapy. [^89^Zr]Zr-DFO-M9346A was found to be straightforward to radiolabel with Zr-89 after conjugation of desferrioxamine to the antibody. Our PET imaging agent showed excellent in vivo performance delineating FRα-positive tumors with high tumor-to-background ratios. Successful in vivo correlation of the pharmacokinetics between companion diagnostic and the radiolabeled ADC allowed us to perform therapy studies allowing the precision prediction of responders and non-responders in small animal studies. In humans, ADC sensitivity likely depends on FRα expression. Intuitively, one of the next steps should be to use this imaging technology in more advanced and heterogeneous mouse models of ovarian cancer. We are confident that [^89^Zr]Zr-DFO-M9346A could ultimately be a suitable companion diagnostic for IMGN853 cancer therapy, quantitatively assessing the folate receptor expression levels non-invasively in patients.

## Additional file

Additional file 1: **Figure S1.** Size exclusion chromatograms of (**a**) M9346A and (**b**) DFO-M9346A. (PDF 279 kb)

Additional file 2: **Figure S2.** Lindmo immunoreactivity assay for ^89^Zr-labeled DFO-M9346A. (PDF 280 kb)

Additional file 3: **Figure S3.** Stability study in human plasma of [^89^Zr]Zr-DFO-M9346A. (PDF 280 kb)

Additional file 4: **Figure S4.** Serial PET imaging at 4 h, 24 h, 48 h, and 72 h post-administration of [^89^Zr]Zr-DFO-M9346A in KB tumor bearing mice. (PDF 335 kb)

Additional file 5: **Figure S5.** Ex vivo biodistribution study at 24 h and 72 h post-injection of [^89^Zr]Zr-DFO-M9346A in KB tumor bearing mice. (PDF 335 kb)

Additional file 6: **Figure S6.** Ex vivo biodistribution study at 24 h and 48 h post-injection of [^89^Zr]Zr-DFO-M9346A in OV90 tumor bearing mice. (PDF 335 kb)

Additional file 7: **Figure S7.** Side-by-side comparison of the ex vivo biodistribution at 48 h post-injection of [^89^Zr]Zr-DFO-M9346A and [^131^I]-IMGN853 in OV90 tumor bearing mice. (PDF 102 kb)

Additional file 8: **Figure S8.** Weights of individual mouse over time during treatment. (PDF 280 kb)

## Abbreviations

ADC: Antibody-drug conjugate
CT: Computed tomography
DFO: Deferoxamine
FRα: Folate receptor alpha
HPLC: High-performance liquid chromatography
mAb: Humanized monoclonal antibody
MSKCC: Memorial Sloan Kettering Cancer Center
PBS: Phosphate buffered saline
PET: Positron emission tomography
