# Supplementary figures and images for: Leveraging PET to image folate receptor α therapy of an antibody-drug conjugate
Source: EJNMMI Res. 2018 Aug 28;8:87. doi: 10.1186/s13550-018-0437-x (PMC6113196; doi:10.1186/s13550-018-0437-x)

**a**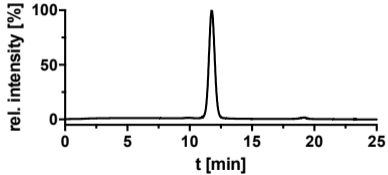**b**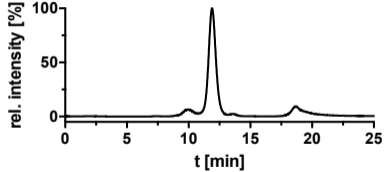

Supplement: Supplementary file 1 — Figure S1. Size exclusion chromatograms of (a) M9346A and (b) DFO-M9346A. (PDF 279 kb) [file 13550_2018_437_MOESM1_ESM.pdf]

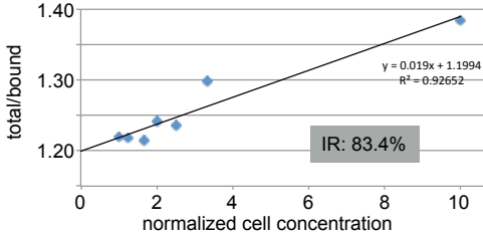

Supplement: Supplementary file 2 — Figure S2. Lindmo immunoreactivity assay for 89Zr-labeled DFO-M9346A. (PDF 280 kb) [file 13550_2018_437_MOESM2_ESM.pdf]

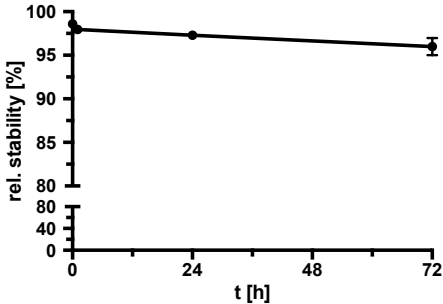

Supplement: Supplementary file 3 — Figure S3. Stability study in human plasma of [89Zr]Zr-DFO-M9346A. (PDF 280 kb) [file 13550_2018_437_MOESM3_ESM.pdf]

M3 (4 h)

M3 (24 h)

M3 (48 h)

M3 (72 h)

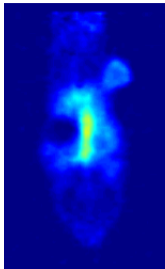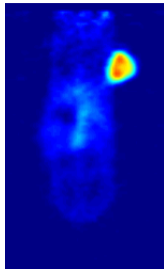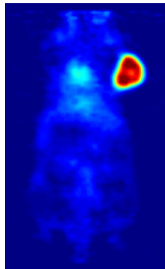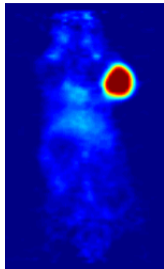

30  
0  
%ID/g

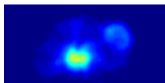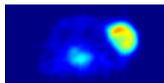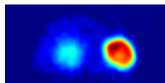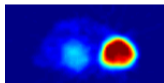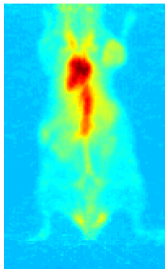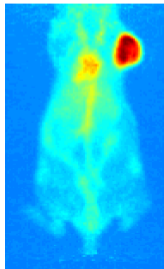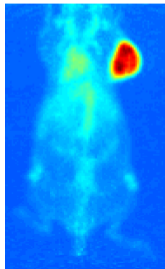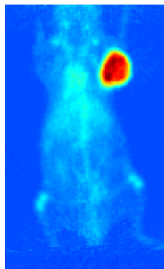

Max  
Min

Supplement: Supplementary file 4 — Figure S4. Serial PET imaging at 4 h, 24 h, 48 h, and 72 h post-administration of [89Zr]Zr-DFO-M9346A in KB tumor bearing mice. (PDF 335 kb) [file 13550_2018_437_MOESM4_ESM.pdf]

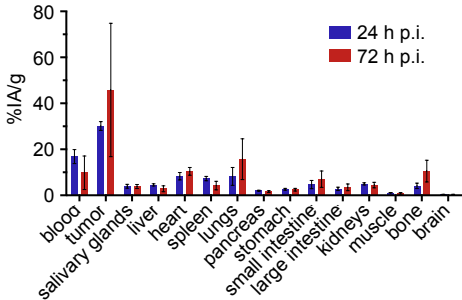

Supplement: Supplementary file 5 — Figure S5. Ex vivo biodistribution study at 24 h and 72 h post-injection of [89Zr]Zr-DFO-M9346A in KB tumor bearing mice. (PDF 335 kb) [file 13550_2018_437_MOESM5_ESM.pdf]

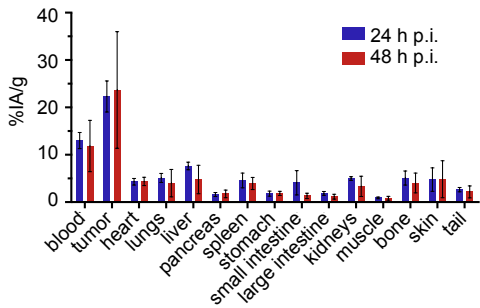

Supplement: Supplementary file 6 — Figure S6. Ex vivo biodistribution study at 24 h and 48 h post-injection of [89Zr]Zr-DFO-M9346A in OV90 tumor bearing mice. (PDF 335 kb) [file 13550_2018_437_MOESM6_ESM.pdf]

■  $[^{89}\text{Zr}]\text{Zr-DFO-M9346A}$   
■  $[^{131}\text{I}]\text{-IMGN853}$

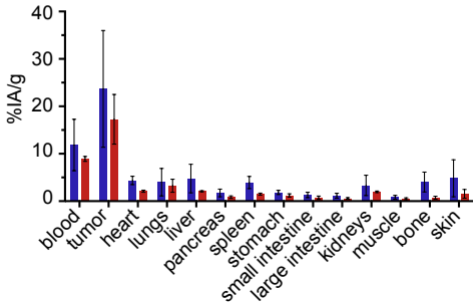

Supplement: Supplementary file 7 — Figure S7. Side-by-side comparison of the ex vivo biodistribution at 48 h post-injection of [89Zr]Zr-DFO-M9346A and [131I]-IMGN853 in OV90 tumor bearing mice. (PDF 102 kb) [file 13550_2018_437_MOESM7_ESM.pdf]

■  $[^{89}\text{Zr}]\text{Zr-DFO-M9346A}$

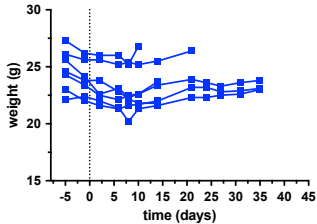

●  $[^{89}\text{Zr}]\text{Zr-DFO-M9346A}$   
+ IMGN853

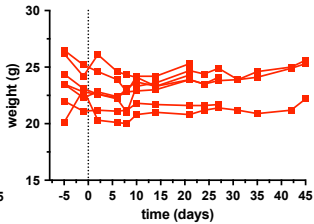

▲ PBS

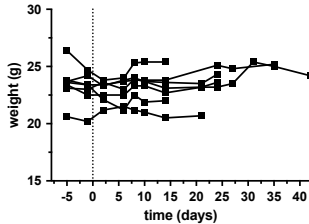

Supplement: Supplementary file 8 — Figure S8. Weights of individual mouse over time during treatment. (PDF 280 kb) [file 13550_2018_437_MOESM8_ESM.pdf]
